# Exploring Cardiac Rehabilitation Barriers Across Health Literacy Levels

**DOI:** 10.3928/24748307-20241127-02

**Published:** 2025-04

**Authors:** Ana Paula Delgado Bomtempo, Gabriela Lima de Melo Ghisi

## Abstract

This study aimed to explore the barriers to cardiac rehabilitation (CR) participation across individuals with different levels of health literacy. A cross-sectional study was conducted among individuals referred to a CR program. Participants completed online surveys assessing CR barriers using the Cardiac Rehabilitation Barriers Scale and health literacy using the Brief Health Literacy Screening Tool. Descriptive statistics and comparisons were performed. Among 881 individuals invited, 400 responded, with varying levels of health literacy: 22(6%) limited, 305(76%) marginal, and 72(18%) adequate. The greatest barriers included family responsibilities, lack of energy, and a preference for managing health alone. However, distinct patterns emerged based on health literacy levels, with individuals citing different barriers. Although no significant differences were observed in CR barriers based on health literacy, understanding individual-specific challenges is crucial for intervention development. Addressing common barriers such as family responsibilities and logistical challenges could enhance CR engagement and adherence. [***HLRP: Health Literacy Research and Practice*. 2025;9(2):e72–e77.**]

Cardiovascular diseases (CVD) remain the leading cause of both morbidity and mortality worldwide, posing significant challenges in health, disability, and economic burden (Vaduganathan et al., 2022). Cardiac rehabilitation (CR) stands out as a well-established and comprehensive secondary prevention strategy capable of mitigating this burden (Anderson et al., 2016). Despite its benefits, CR is grossly underutilized globally (Grace et al., 2021) due to multifaceted barriers, including lack of awareness at the health system level, insufficient referrals by of providers, long program wait times and logistical factors and time conflicts faced by patients (Grace et al., 2021).

Considerable research has delved into sex differences in CR barriers, (Ghisi et al., 2023) as well as age, (Grace et al., 2009) ethnicity (Garfein et al., 2022), and socioeconomic status (Shanmugasegaram et al., 2012). However, while several studies have investigated the relation between CR and health literacy levels (Aaby et al., 2020; Beauchamp et al., 2020; Isselhard et al., 2022; Lunde et al., 2024) there is sparse data examining the barriers to CR among different levels of health literacy. To our knowledge, no study has specifically investigated CR barriers across varying levels of health literacy. Health literacy, defined as the degree to which an individual can access, process, and comprehend basic health information and services to inform and participate in health decisions (Centers for Disease Control and Prevention, n.d.), holds a critical and decisive role in health care delivery and outcomes in secondary prevention of CVDs (Magnani et al., 2018). Understanding the impact of health literacy on CR barriers is essential for developing tailored interventions and improving access to rehabilitation services. Therefore, this study aimed to explore CR barriers across individuals with different health literacy levels.

## Methods

This was a cross-sectional study, where participation in the survey was voluntary, and responses were kept confidential. Individuals referred to the CR program at the University Health Network in Canada between June 2023 and February 2024, proficient in reading English, and with a registered email address in the hospital database were invited to complete online surveys. Data collection occurred through an online survey administered via REDCap. Approval for the study was granted as a quality improvement project by the local Research Ethics Board (QIRC 23-0545). Participants provided implied consent by completing the survey.

Well-established and externally validated tools were used to assess barriers to CR participation and health literacy. The Cardiac Rehabilitation Barriers Scale (CRBS) was used to assess barriers to CR (Shanmugasegaram et al., 2012). It consists of 21 statements where participants rated their level of agreement on a 5-point Likert-type scale ranging from 1 = *strongly disagree* to 5 = *strongly agree*. It is comprised of four subscales, namely: lack of perceived need, unmet CR preferences, work/family/time conflicts, and logistical factors (e.g., distance, cost, and including clinical issues such as comorbidities, as well as health care system issues like lack of referral). Mean scores were computed, with higher scores indicating greater barriers to patient enrollment/participation in CR.

Health literacy was assessed using the Brief Health Literacy Screening Tool (Haun et al., 2012), which includes four items rated on a 1-to-5-point scale based on participant responses. Scores ranged from 4 to 20, with classifications of limited health literacy (LHL; 4 to 12 points), marginal health literacy (MHL; 13 to 16 points), and adequate health literacy (AHL; 17 to 20 points). Data were exported to SPSS 28, and a descriptive analysis of CRBS and BRIEF scores was performed. A one-way ANOVA was conducted to compare CRBS scores across the health literacy levels.

## Results

Overall, 881 individuals were invited to complete the surveys, and 400 (45%) responses were received. Of these, 22 (6%) had LHL, 305 (76%) had MHL, and 72 (18%) AHL.

**Table 1** presents the characteristics of participants, overall and by health literacy levels. Participants with LHL had a mean age of 69.4 ± 10.2 years and were predominantly non-White (48%). Those with MHL had a mean age of 65.5 ± 12.4 years and were mostly White (48%). Conversely, participants with AHL age 67 ± 12.7 years and were also mostly White (57%). Across all health literacy levels, there was a high proportion of individuals who were men, employed, and with high education (i.e., a university degree). Coronary artery bypass grafting, followed by percutaneous coronary intervention, were the most frequent reasons for referral to the CR program.

**Table 1:**
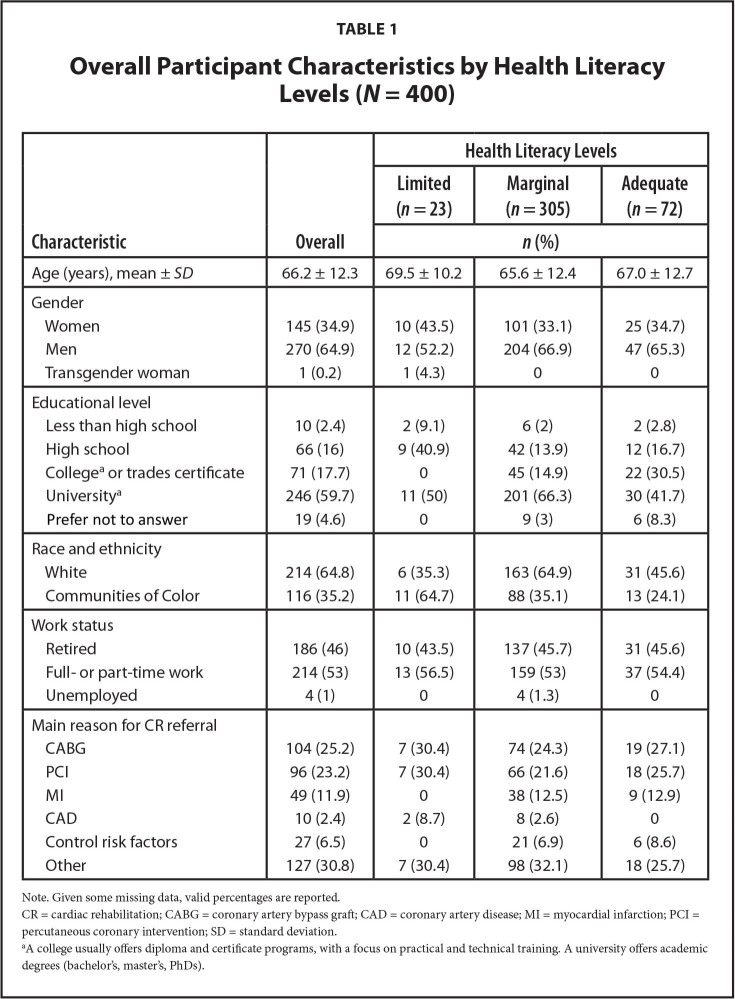
Overall Participant Characteristics by Health Literacy Levels (*N* = 400)

**Characteristic**	**Overall**	**Health Literacy Levels**		

		**Limited (*n* = 23)**	**Marginal (*n* = 305)**	**Adequate (*n* = 72)**

		***n* (%)**		

Age (years), mean ± *SD*	66.2 ± 12.3	69.5 ± 10.2	65.6 ± 12.4	67.0 ± 12.7

Gender				
Women	145 (34.9)	10 (43.5)	101 (33.1)	25 (34.7)
Men	270 (64.9)	12 (52.2)	204 (66.9)	47 (65.3)
Transgender woman	1 (0.2)	1 (4.3)	0	0

Educational level				
Less than high school	10 (2.4)	2 (9.1)	6 (2)	2 (2.8)
High school	66 (16)	9 (40.9)	42 (13.9)	12 (16.7)
College^a^ or trades certificate	71 (17.7)	0	45 (14.9)	22 (30.5)
University^a^	246 (59.7)	11 (50)	201 (66.3)	30 (41.7)
Prefer not to answer	19 (4.6)	0	9 (3)	6 (8.3)

Race and ethnicity				
White	214 (64.8)	6 (35.3)	163 (64.9)	31 (45.6)
Communities of Color	116 (35.2)	11 (64.7)	88 (35.1)	13 (24.1)

Work status				
Retired	186 (46)	10 (43.5)	137 (45.7)	31 (45.6)
Full- or part-time work	214 (53)	13 (56.5)	159 (53)	37 (54.4)
Unemployed	4 (1)	0	4 (1.3)	0

Main reason for CR referral				
CABG	104 (25.2)	7 (30.4)	74 (24.3)	19 (27.1)
PCI	96 (23.2)	7 (30.4)	66 (21.6)	18 (25.7)
MI	49 (11.9)	0	38 (12.5)	9 (12.9)
CAD	10 (2.4)	2 (8.7)	8 (2.6)	0
Control risk factors	27 (6.5)	0	21 (6.9)	6 (8.6)
Other	127 (30.8)	7 (30.4)	98 (32.1)	18 (25.7)

Note. Given some missing data, valid percentages are reported.

CR = cardiac rehabilitation; CABG = coronary artery bypass graft; CAD = coronary artery disease; MI = myocardial infarction; PCI = percutaneous coronary intervention; SD = standard deviation.

a A college usually offers diploma and certificate programs, with a focus on practical and technical training. A university offers academic degrees (bachelor's, master's, PhDs).

All individuals acknowledged family responsibilities as a significant barrier to CR. Additionally, the LHL group claimed distance as another great barrier (i.e., with the highest CRBS score), while the MHL group also claimed lack of energy as a great barrier to CR participation. Moreover, logistical factors were consistently identified as an important barrier across all health literacy levels. The lowest barriers identified (i.e., with the lowest CRBS scores) were related to costs and perceived need/health care factors, specifically related to certain misconceptions about the effectiveness of CR (“Many people with heart problems don't go, and they are fine”) and personal exercise habits (“I already exercise at home, or in my community”). As indicated in **Table 2**, there were no significant differences in CRBS scores based on health literacy, both overall and across subscales and individual items.

**Table 2:**
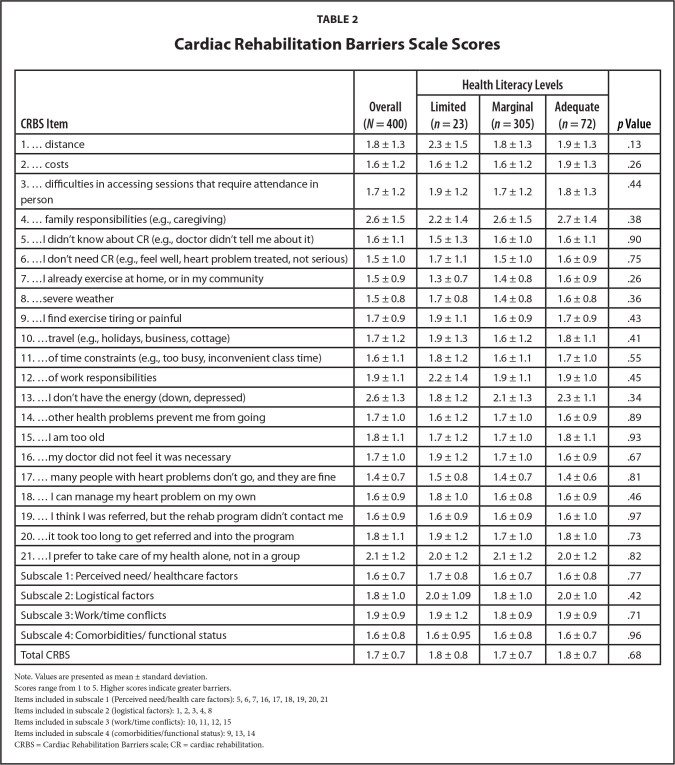
Cardiac Rehabilitation Barriers Scale Scores

**CRBS Item**	**Overall (*N* = 400)**	**Health Literacy Levels**			***p* Value**
		**Limited (*n* = 23)**	**Marginal (*n* = 305)**	**Adequate (*n* = 72)**	
1. … distance	1.8 ± 1.3	2.3 ± 1.5	1.8 ± 1.3	1.9 ± 1.3	.13
2. … costs	1.6 ± 1.2	1.6 ± 1.2	1.6 ± 1.2	1.9 ± 1.3	.26
3. … difficulties in accessing sessions that require attendance in person	1.7 ± 1.2	1.9 ± 1.2	1.7 ± 1.2	1.8 ± 1.3	.44
4. … family responsibilities (e.g., caregiving)	2.6 ± 1.5	2.2 ± 1.4	2.6 ± 1.5	2.7 ± 1.4	.38
5. …I didn't know about CR (e.g., doctor didn't tell me about it)	1.6 ± 1.1	1.5 ± 1.3	1.6 ± 1.0	1.6 ± 1.1	.90
6. …I don't need CR (e.g., feel well, heart problem treated, not serious)	1.5 ± 1.0	1.7 ± 1.1	1.5 ± 1.0	1.6 ± 0.9	.75
7. …I already exercise at home, or in my community	1.5 ± 0.9	1.3 ± 0.7	1.4 ± 0.8	1.6 ± 0.9	.26
8. …severe weather	1.5 ± 0.8	1.7 ± 0.8	1.4 ± 0.8	1.6 ± 0.8	.36
9. …I find exercise tiring or painful	1.7 ± 0.9	1.9 ± 1.1	1.6 ± 0.9	1.7 ± 0.9	.43
10. …travel (e.g., holidays, business, cottage)	1.7 ± 1.2	1.9 ± 1.3	1.6 ± 1.2	1.8 ± 1.1	.41
11. …of time constraints (e.g., too busy, inconvenient class time)	1.6 ± 1.1	1.8 ± 1.2	1.6 ± 1.1	1.7 ± 1.0	.55
12. …of work responsibilities	1.9 ± 1.1	2.2 ± 1.4	1.9 ± 1.1	1.9 ± 1.0	.45
13. …I don't have the energy (down, depressed)	2.6 ± 1.3	1.8 ± 1.2	2.1 ± 1.3	2.3 ± 1.1	.34
14. …other health problems prevent me from going	1.7 ± 1.0	1.6 ± 1.2	1.7 ± 1.0	1.6 ± 0.9	.89
15. …I am too old	1.8 ± 1.1	1.7 ± 1.2	1.7 ± 1.0	1.8 ± 1.1	.93
16. …my doctor did not feel it was necessary	1.7 ± 1.0	1.9 ± 1.2	1.7 ± 1.0	1.6 ± 0.9	.67
17. … many people with heart problems don't go, and they are fine	1.4 ± 0.7	1.5 ± 0.8	1.4 ± 0.7	1.4 ± 0.6	.81
18. … I can manage my heart problem on my own	1.6 ± 0.9	1.8 ± 1.0	1.6 ± 0.8	1.6 ± 0.9	.46
19. … I think I was referred, but the rehab program didn't contact me	1.6 ± 0.9	1.6 ± 0.9	1.6 ± 0.9	1.6 ± 1.0	.97
20. …it took too long to get referred and into the program	1.8 ± 1.1	1.9 ± 1.2	1.7 ± 1.0	1.8 ± 1.0	.73
21. …I prefer to take care of my health alone, not in a group	2.1 ± 1.2	2.0 ± 1.2	2.1 ± 1.2	2.0 ± 1.2	.82
Subscale 1: Perceived need/healthcare factors	1.6 ± 0.7	1.7 ± 0.8	1.6 ± 0.7	1.6 ± 0.8	.77
Subscale 2: Logistical factors	1.8 ± 1.0	2.0 ± 1.09	1.8 ± 1.0	2.0 ± 1.0	.42
Subscale 3: Work/time conflicts	1.9 ± 0.9	1.9 ± 1.2	1.8 ± 0.9	1.9 ± 0.9	.71
Subscale 4: Comorbidities/functional status	1.6 ± 0.8	1.6 ± 0.95	1.6 ± 0.8	1.6 ± 0.7	.96
Total CRBS	1.7 ± 0.7	1.8 ± 0.8	1.7 ± 0.7	1.8 ± 0.7	.68

Note. Values are presented as mean ± standard deviation.

Scores range from 1 to 5. Higher scores indicate greater barriers.

Items included in subscale 1 (Perceived need/health care factors): 5, 6, 7, 16, 17, 18, 19, 20, 21

Items included in subscale 2 (logistical factors): 1, 2, 3, 4, 8

Items included in subscale 3 (work/time conflicts): 10, 11, 12, 15

Items included in subscale 4 (comorbidities/functional status): 9, 13, 14

CRBS = Cardiac Rehabilitation Barriers scale; CR = cardiac rehabilitation.

## Discussion

This study represents a pioneering effort in investigating barriers to CR across individuals with varying levels of health literacy. While no significant difference was observed in the total score when comparing CR barriers among individuals with different health literacy levels, our findings shed light on the pervasive challenge of “family responsibility” across all participant groups. This common barrier underscores the complex interplay between personal obligations and health care engagement, suggesting the need for tailored interventions that address familial and caregiving responsibilities to enhance CR participation and outcomes.

In addition to family responsibilities, individuals with LHL underscored challenges related to distance as important barriers to engaging in CR. This highlights the significant impact of geographical accessibility on their ability to participate in programs. Virtual CR has emerged as a new model that can address distance barriers (Beatty et al., 2023). However, it also comes with implicit biases that may disproportionately affect those susceptible to low health literacy, as it requires access to a smartphone, stable internet, and often a stable housing situation, as well as access to wearable devices (Estacio et al., 2017). In this context, ensuring accessibility and ease of understanding instructions becomes paramount, particularly for individuals with LHL (Barksdale et al., 2023). Therefore, interventions and educational materials should be developed in a way that patients can easily access and comprehend, addressing both geographical and literacy-related barriers to CR participation.

Among participants with MHL and AHL, lack of energy (i.e., feeling down or depressed) also emerged as a significant obstacle to CR participation. In this context, it is crucial to first understand the underlying reasons for the reported low energy among these individuals. This may stem from the additional cognitive and emotional burdens faced by individuals with low health literacy levels (Serper et al., 2014), which can exacerbate feelings of fatigue and overwhelm. Future interventions should focus on tailoring educational resources and support to address the unique challenges faced by individuals with low health literacy levels by developing strategies that foster self-efficacy in managing energy levels within the context of CR, rather than merely suggesting additional physical activity or dietary changes. Flexible program schedules and motivational strategies may also be vital in enhancing participation in CR for this group (Everett et al., 2021; Rouleau et al., 2018).

Previous studies using the CRBS conducted in countries such as Portugal, Greece, South Korea, Iran, Brazil, and the United States have reported varying scores related to CR participation, with many studies indicating high barriers to participation, typically scoring above 4 out of 5 (Stewart et al., 2023). In contrast, the current study presents general scores lower than 3 out of 5, suggesting that the specific cohort studied may be more favorable for CR participation compared to previous research findings in other regions. Despite the differences in overall scores, the types of barriers identified across studies remain similar. Common obstacles include issues related to accessibility, patient knowledge, and psychological factors (Stewart et al., 2023). This consistency highlights the persistent challenges faced by patients in accessing CR, regardless of geographical location, and underscores the importance of addressing these barriers to improve participation rates in CR programs globally.

Health literacy significantly influences the successful management of CVD (Ghisi et al., 2018). Patients with LHL may face challenges in comprehending, engaging with, and actively participating in CR, potentially leading to missed opportunities for both physiological and psychosocial benefits associated with participation (Magnani et al., 2018). It is important to recognize that individuals referred to and attending CR programs are already a selected group, typically motivated to engage in their health management (Shao et al., 2023). In comparison to other studies using the similar health literacy questionnaires, the current study population exhibits a relatively higher proportion of individuals with marginal health literacy and a lower proportion of those with inadequate health literacy, suggesting that this cohort is overall more health literate than populations previously studied in the context of CR (Ghisi et al., n.d.). This higher baseline of health literacy may reflect the unique characteristics of the group, yet the challenges faced by individuals with lower health literacy remain significant and warrant targeted interventions.

Patient education strategies could be used to mitigate the impact of LHL on CR outcomes (Shao et al., 2023). These strategies often include adopting a user-centered design approach for CR materials, simplifying communication to ensure clarity, and actively confirming understanding with all participants. Various interventions have been applied in CR programs to improve outcomes for patients with LHL. For instance, using visual aids, plain language, and Teach-Back methods to confirm understanding have proven effective in enhancing comprehension and engagement (Beauchamp et al., 2022).

Additionally, personalized educational tools like digital platforms tailored to individual literacy levels have shown promise in improving adherence and outcomes (Carson et al., 2024; Ghisi et al., 2024). Our study adds to these efforts by highlighting the specific challenges faced by individuals MHL and LHL and reinforcing the need for targeted strategies that address both comprehension and engagement barriers. This further emphasizes the necessity of tailored educational interventions and supports the ongoing development of patient-centered, accessible CR programs that take health literacy into account.

## Limitations

While our study offers valuable insights into the barriers to CR participation among individuals with varying health literacy levels, several limitations should be acknowledged. Firstly, the cross-sectional design of the study limits our ability to establish causal relationships. Additionally, our sample consisted of patients referred to a single CR program, which may not be representative of the broader population. We were also unable to confirm the CR participation of this sample. Moreover, the reliance on self-reported data for health literacy and barriers to CR participation introduces the potential for recall and social desirability biases. Furthermore, the assessment of health literacy was limited to one questionnaire and did not include an assessment of digital health literacy, potentially overlooking an important aspect of patients' ability to engage with CR programs. Moreover, the majority of our sample comprised individuals with MHL levels, which could have skewed the findings. Additionally, there is potential for selection bias, as the inclusion criteria required participants to be proficient in reading English and have a registered email address. This criterion automatically excluded non-English speaking patients, who are at higher risk for low health literacy, as well as individuals who may be unhoused or lack the financial means for internet access, potentially limiting the generalizability of our findings.

Future studies should employ multi-site designs with larger, diverse samples and comprehensive assessments of health literacy, including digital health literacy. Additionally, assessing key characteristics such as geographic information, native status or immigration status, socioeconomic status, and insurance coverage will enhance understanding of CR barriers.

## Conclusion

In conclusion, our study sheds light on the complex landscape of barriers to CR participation, highlighting the significant role of health literacy in shaping these barriers. While we found no significant differences in CR barriers across different health literacy levels, our findings underscore the importance of addressing individual-specific barriers to enhance engagement and adherence to CR programs. The identification of family responsibilities, lack of energy, and logistical challenges as prominent barriers emphasizes the need for tailored interventions to overcome these obstacles. Moving forward, efforts to develop comprehensive interventions and educational materials that are accessible and understandable to all patients, regardless of their health literacy level, will be essential in promoting equitable access to CR and improving outcomes for individuals with CVD.
